# Anti-Inflammatory and Antitumor Effects of Hydroxytyrosol but Not Oleuropein on Experimental Glioma In Vivo. A Putative Role for the Renin-Angiotensin System

**DOI:** 10.3390/biomedicines6010011

**Published:** 2018-01-26

**Authors:** María Jesús Ramírez-Expósito, José Manuel Martínez-Martos

**Affiliations:** Experimental and Clinical Physiopathology Research Group CTS1039, Department of Health Sciences, School of Health Sciences, University of Jaén, Campus Universitario Las Lagunillas, E23071 Jaén, Spain; jmmartos@ujaen.es

**Keywords:** oleuropein, hydroxytyrosol, glioma, C6, renin-angiotensin system, aminopeptidase, inflammation

## Abstract

Functional roles of the angiotensin peptides of the renin-angiotensin system (RAS) cascade can be analyzed through their corresponding proteolytic regulatory enzymes aspartyl aminopeptidase (ASAP), aminopeptidase A (APA), aminopeptidase B (APB), aminopeptidase N (APN) and insulin-regulated aminopeptidase (IRAP). These enzyme activities generate active or inactive angiotensin peptides that alter the ratios between their bioactive forms, regulating several important processes such as the regulation of cardiovascular functions, body water regulation, normal memory consolidation and retrieval, but also cell growth, differentiation and apoptosis or the inflammatory response. We have previously described that the treatment with hydroxytyrosol but not with oleuropein or with the mixture of both compounds led to the significant inhibition of tumor growth in an in vivo glioma model by mechanisms not only related to redox balance. Using this glioma model, here we analyze the effects of the phenolic compounds oleuropein and hydroxytyrosol in circulating RAS-regulating ASAP, APA, APN, APB and IRAP specific activities and the pro-inflammatory cytokines IL-6 and TNFα to understand the relationship between the antitumor and anti-inflammatory effects of hydroxytyrosol, but not oleuropein, and the components of the RAS. We found that oleuropein increased all the activities analyzed and promoted a pro-inflammatory status, whereas hydroxytyrosol only modified ASAP and IRAP activities and promotes an anti-inflammatory status. When administrated together, oleuropein overrode the effects of hydroxytyrosol. Our results suggest a role for angiotensin III and angiotensin 1-7 in both tumor growth inhibition and anti-inflammatory response promoted by hydroxytyrosol.

## 1. Introduction

The inflammatory response plays a crucial role in neoplastic diseases and closely correlates with tumor progression and metastasis. In fact, the severity of inflammation serves as an important indicator of tumor progression and survival among patients with cancer. Furthermore, neoplastic cells often over-express pro-inflammatory mediators, including proteases, eicosanoids, cytokines, and chemokines. Several cytokines, such as tumor necrosis factor (TNF)-α, interleukins (IL-6, IL-17, IL-12, IL-23, IL-10), transforming growth factor (TGF)-β, and macrophage migration inhibitory factor (MIF) have been linked with both experimental and human cancers [1,2]. Renin-angiotensin system (RAS) components are classically related to an increase in the inflammatory response by the induction of the expression of several cytokines, transcription factors and cell adhesion molecules [3,4], among other multiple much better known physiological processes such as the regulation of cardiovascular functions, body water regulation, normal memory consolidation and retrieval [5], cell growth, cell differentiation and apoptosis [6]. In the central nervous system (CNS), it has been suggested that several angiotensin peptides may be involved in essential steps in both brain tumor infiltration and in brain tumor-associated angiogenesis. Furthermore, it has been also shown that some neurons or glial cells and glioma cells express the components of the RAS and their receptors, and although their functions are unclear, some of them seem to be related to inflammatory processes [5,7,8,9]. We have previously described that angiotensin peptides are involved in tumor growth in the rat model of C6 gliomas implanted at the subcutaneous region, and that their role can be analyzed through their corresponding proteolytic regulatory enzymes [10]. In the RAS system, the first enzyme involved is the acid aspartate-protease renin [11], which converts angiotensinogen to angiotensin I (AngI). This angiotensin is inactive, and is converted to active angiotensin II (AngII) by the dipeptidilcarboxypeptidase angiotensin-converting enzyme (ACE-1) [12]. Through a similar mechanism of AngII metabolism are produced active angiotensin III (AngIII) and angiotensin IV (AngIV), with the involvement of angiotensinases of aminopeptidase-type. Angiotensinases are peptidases that generate active or inactive angiotensin peptides that alter the ratios between their bioactive forms. To this category belong aspartyl aminopeptidase (ASAP) and aminopeptidase A (APA) [13], aminopeptidase B (APB) and aminopeptidase N (APN), which are involved in the removal of the first amino acid of the peptide chain of AngII for the formation of AngIII and AngIV. Thus, AngII degradation begins with the action of APA, which removes the N-terminal Asp to produce AngIII. AngIII is further converted to AngIV by APN or APB. AngII and AngIII mediates their action through AT_1_ and AT_2_ receptor subtypes [14,15], whereas AngIV seems to act at the AT_4_ receptor subtype [4], which has been identified as the enzyme insulin-regulated aminopeptidase (IRAP) or, more recently, as the hepatocyte growth factor (HGF)/c-Met receptor system [5]. On the other hand, tissue carboxypeptidases and other proteolytic enzymes (such as trypsin, chymotrypsin, pepsin and others) contribute to the inactivation of the active forms of angiotensins, transforming them into inactive fragments and amino acid constituents [4,12]. A separate pathway for the synthesis of AngIII, independent of AngII formation, is via the nonapeptide [des-Asp1]AngI, formed from AngI by ASAP [16]. This nonapeptide is converted directly to AngIII via ACE-1. A third alternative route involves the ACE-1 homolog ACE-2 [17], which promotes AngII degradation to the heptapeptide Ang1-7. The majority of the reported effects of Ang1-7 are mediated via the activation of a G-protein coupled receptor, the Mas receptor [18]. ASAP and APA inactivate Ang1-7, converting it into Ang2-7. It has been described that the ACE-2/Ang1-7/Mas cascade opposes the effects of the ACE-1/AngII/AT_1_ receptor axis.

Phenolic compounds derived from olives and virgin olive oils, mainly oleuropein and its major metabolite hydroxytyrosol, exert important anti-inflammatory, cardioprotective and anticancer activities both in vitro and in vivo due to their antioxidant properties, reducing the risk of mutagenesis and carcinogenesis [19]. In a previous report [20], we showed that the treatment with hydroxytyrosol, but not with oleuropein or with the mixture of both compounds, led to the significant inhibition of tumor growth in an in vivo glioma model through mechanisms involving endogenous enzymatic and non-enzymatic antioxidant defense systems, as demonstrated by the decreases in oxidative stress biomarkers, such as TBARS and protein oxidation. Thus, the hydroxytyrosol treatment maintained the nonenzymatic antioxidant defense systems similar to those in the healthy animals and positively modified the enzymatic antioxidant defense systems. In contrast, oleuropein did not possess these antitumor effects and even promoted tumor growth despite being a more potent antioxidant than hydroxytyrosol, supporting the notion that modification of antioxidant defense systems is an additional effect of hydroxytyrosol, which may act as an antitumor compound through other unknown mechanisms. In the present report, we analyze the effects of phenolic compounds oleuropein, hydroxytyrosol and a mixture of both in circulating RAS-regulating ASAP, APA, APN, APB and IRAP specific activities together to the proinflammatory cytokines TNF-α and IL-6 in an animal model of glioma to better understand the relationship between RAS, inflammation and tumor growth in this experimental model.

## 2. Experimental Section

### 2.1. Cell Culture

C6 rat glioma cell line was obtained from the American Type Culture Collection (No. CCL-107) (ATTC; Manassas, VA, USA) and cultured in Dulbecco’s Modified Eagle Medium/HAM F-12 mixture (Sigma-Aldrich, Madrid, Spain), supplemented with 5% fetal bovine serum (FBS), without antibiotics. Cells were incubated at 37 °C in a modified atmosphere of 5% CO_2_/95% air until confluence. Absence of mycoplasma contamination was assessed regularly using Hoechst 33258 staining (Invitrogen, Madrid, Spain).

### 2.2. Animals and Treatments

Thirty-two male adult Wistar rats (350 ± 3.24 g body weight) were used in this study. The animals were provided by Harlan Ibérica S.A. and maintained at the animal house of the University of Jaen in a controlled environment at a constant temperature (25 °C) with a 12 h-light/12 h-dark cycle. The rats were housed in cages and given free access to standard laboratory rat food and water. The experimental procedures for animal use and care were in accordance with the European Community Council directive (2010/63/EU). The protocols were approved by the Bioethical Committee of the University of Jaen (reference # PEJA 4957M; 28/8/2014). The animals were randomly divided into four groups of eight rats each. Animals were subjected to C6 glioma cell implantation (see below). Ten days after C6 glioma cell implantation, animals in three of the tumor groups received daily subcutaneous injections of 100 µg oleuropein (Cymit Química S.L., Barcelona, Spain), 100 µg hydroxytyrosol (Cymit Química S.L., Barcelona, Spain) or 100 µg oleuropein plus 100 µg hydroxytyrosol dissolved in 500 µL saline solution for 5 days. The other tumor group (control group) received vehicle-only injections (saline solution) for the same time period.

### 2.3. Implantation of C6 Glioma Cells

Five million C6 glioma cells suspended in 25 µL of culture medium without FBS were injected subcutaneously in both dorsal flanks of the rats using a Hamilton syringe with a 26-gauge needle. The characteristics of this glioma model have been previously described [10,20].

### 2.4. Measurement of Tumor Volume and Sample Collection

The size of the abdominal tumors was measured with slide calipers at the end of the treatments. The tumor volume was defined as 1⁄2(*a* × *b*)^2^ (a: long diameter and b: short diameter). At the end of the treatments, the rats were anesthetized with equitensin (2 mL/kg body weight) by intraperitoneal injection. Blood samples, which were obtained from the left cardiac ventricle, were drawn into tubes without anticoagulant, allowed to clot, and then centrifuged for 10 min at 3000× *g* to obtain the serum, which was frozen and stored at −80 °C until use.

### 2.5. Aminopeptidase Activity Assays

#### 2.5.1. Aspartyl Aminopeptidase (ASAP) Activity Assay

ASAP was measured fluorometrically using aspartyl-ß-naphthylamide (AspNNap) as the substrate (Sigma-Aldrich, Madrid, Spain). Briefly, 10 µL of each sample was incubated in triplicate for 30 min at 37 °C with 100 µL of the substrate solution containing 100 μM AspNNap, 1.3 μM ethylenediaminetetraacetic acid (EDTA) and 2 mM MnCl_2_ in 50 mM of phosphate buffer, pH 7.4.

#### 2.5.2. Aminopeptidase A (APA) Activity Assay

APA activity was measured in the same way using glutamyl-ß-naphthylamide (GluNNap) as the substrate (Sigma-Aldrich, Madrid, Spain). 10 µL of each sample was incubated in triplicate for 30 min at 37 °C with 100 µL of the substrate solution containing 100 μM GluNNap, 0.65 mM dithiothreitol (DTT) and 50 mM CaCl_2_ in 50 mM of phosphate buffer, pH 7.4.

#### 2.5.3. Aminopeptidase N (APN) Activity Assay

APN was measured fluorometrically using alanyl-ß-naphthylamide (AlaNNap) as substrate (Sigma-Aldrich, Madrid, Spain). 10 µL of each sample were incubated by triplicate for 30 min at 37 °C with 100 μL of the substrate solution containing 100 μM of AlaNNap and 0.65 mM dithiothreitol (DTT) in 50 mM phosphate buffer, pH 7.4.

#### 2.5.4. Aminopeptidase B (APB) Activity Assay

APB was measured fluorometrically using arginyl-β-naphthylamide (ArgNNap) as substrate (Sigma-Aldrich, Madrid, Spain). 10 µL of each sample were incubated by triplicate for 30 min at 37 °C with 100 μL of the substrate solution containing 100 μM of ArgNNap and 0.65 mM dithiothreitol (DTT) in 50 mM phosphate buffer, pH 7.4.

#### 2.5.5. Insulin-Regulated Aminopeptidase (IRAP) Activity Assay

IRAP activity was measured fluorometrically using leucyl-ß-naphthylamide (LeuNNap) as substrate (Sigma-Aldrich, Madrid, Spain). 10 µL of each sample were incubated by triplicate for 30 min at 37 °C with 100 μL of the substrate solution containing 100 μM of LeuNNap and 0.65 mM dithiothreitol (DTT) in 50 mM phosphate buffer, pH 7.4.

All the reactions were stopped by adding 100 µL of 0.1 M acetate buffer, pH 4.2. The amount of ß-naphthylamine released as the result of the enzymatic activities was measured fluorometrically at 412 nm emission wavelength with and excitation wavelength of 345 nm. Proteins were quantified also in triplicate by the method of Bradford, using bovine serum albumin (BSA) as standard. Specific enzyme activities were expressed as picomoles of the corresponding aminoacyl-β-naphthylamide hydrolyzed per min per mg of protein, by using a standard curve prepared with the latter compound under corresponding assay conditions. The fluorogenic assay was linear with respect to time of hydrolysis and protein content.

### 2.6. Cytokine Production Assay

ELISA kits were used for determination of pro-inflammatory cytokine concentrations such as IL-6 (R&D Systems, Grontal, Granada, Spain) and TNF-α (BioSource, Camarillo, CA, USA) in the sera samples. Results were calculated against standard curves generated using known amounts of recombinant cytokines in accordance with the manufacturer’s instructions in a microplate reader (Tecan Genios Plus, Tecan Ibérica de Instrumentación, Barcelona, Spain) at a wavelength of 450 nm. Samples were assayed in duplicate.

### 2.7. Statistical Analysis

All values represent the mean ± standard error of the mean (SEM). The data were analyzed by ANOVA plus Newman-Keul’s test using IBM SPSS software (version 19, IBM Corporation, Armonk, NY, USA). Values of *p* < 0.05 were considered significant.

## 3. Results

### 3.1. Effects of Oleuropein and Hydroxytyrosol on Tumor Growth

**Figure 1:**
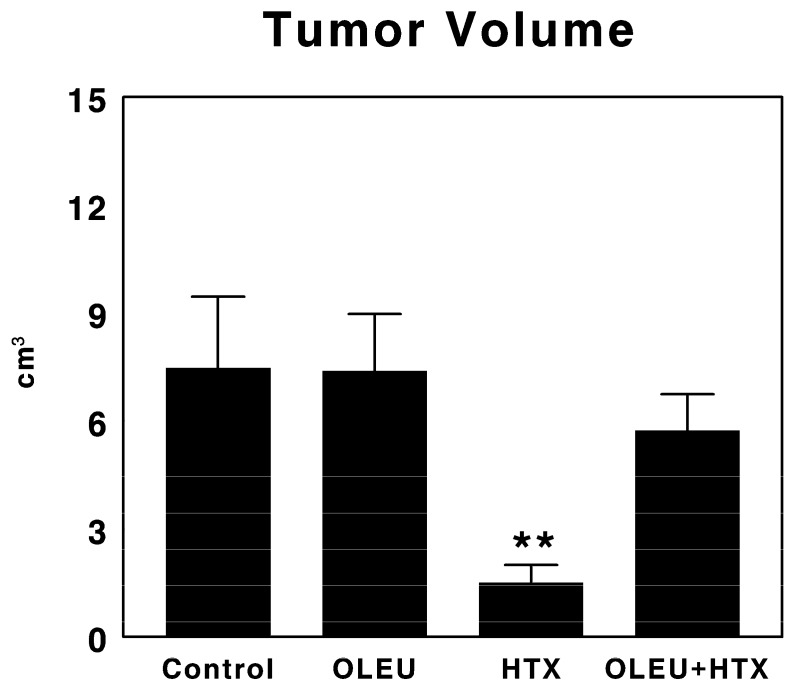
Tumor volumes after the implantation of C6 glioma cells into the subcutaneous region after the treatment with vehicle (Control), 100 µg/d oleuropein (OLEU), 100 µg/d hydroxytyrosol (HTX) and 100 µg oleuropein plus 100 µg of hydroxytyrosol/d (OLEU + HTX) for five days. Results are expressed in cm^3^ (Mean ± SEM; *n* = 8; ** *p* < 0.01).

The effects of the administration of oleuropein, hydroxytyrosol and a mixture of both compounds for five days on tumor growth in our experimental glioma model are presented in Figure 1. The tumor volume in non-treated animals reached 7.47 ± 1.98 cm^3^ over the course of the experiment. Similarly, the animals treated with oleuropein showed a tumor volume of 7.39 ± 1.58 cm^3^). In contrast, those animals treated with hydroxytyrosol showed a significant decrease in tumor volume (*p* < 0.01), reaching only 1.51 ± 0.49 cm^3^). Finally, the animals treated with both oleuropein and hydroxytyrosol showed a tumor volume of 5.73 ± 1.01 cm^3^).

### 3.2. Effects of Oleuropein and Hydroxytyrosol on RAS-Regulating Aminopeptidases

**Figure 2:**
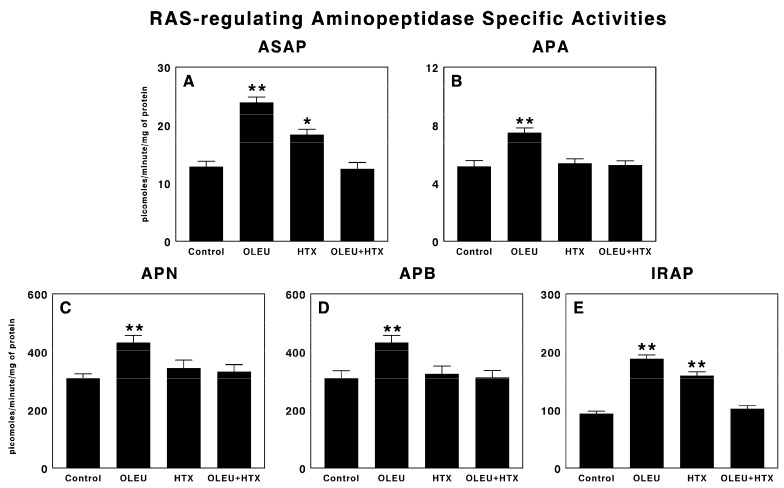
Renin-angiotensin system-regulating (**A**) aspartyl aminopeptidase (ASAP); (**B**) aminopeptidase A (APA); (**C**) aminopeptidase N (APN); (**D**) aminopeptidase B (APB); and (**E**) insulin-regulated aminopeptidase (IRAP) specific activities, in sera of animals with C6 glioma implanted into the subcutaneous region, treated with vehicle (Control), 100 µg/d oleuropein (OLEU), 100 µg/d hydroxytyrosol (HTX) and 100 µg oleuropein plus 100 µg of hydroxytyrosol/d (OLEU + HTX) for five days. Results are expressed in picomoles of the corresponding aminoacyl-ß-naphthylamide hydrolyzed per minute and per mg of protein (Mean ± SEM; *n* = 8; * *p* < 0.05; ** *p* < 0.01).

Figure 2 shows the effects of the administration of oleuropein, hydroxytyrosol and a mixture of both compounds for five days on tumor growth in our experimental glioma model on RAS-regulating aminopeptidases. Oleuropein administration significantly increased (*p* < 0.01) all the specific activities measured (ASAP, APA, APN, APB and IRAP) (Figure 2A–E). However, hydroxytyrosol administration significantly increased ASAP specific activity (*p* < 0.05; Figure 2A) and IRAP specific activity (*p* < 0.01; Figure 2E), but to a lesser degree than oleuropein. The mixture of oleuropein plus hydroxytyrosol did not change any of the specific activities measured (Figure 2A–E).

### 3.3. Effects of Oleuropein and Hydroxytyrosol on Cytokine Production

**Figure 3:**
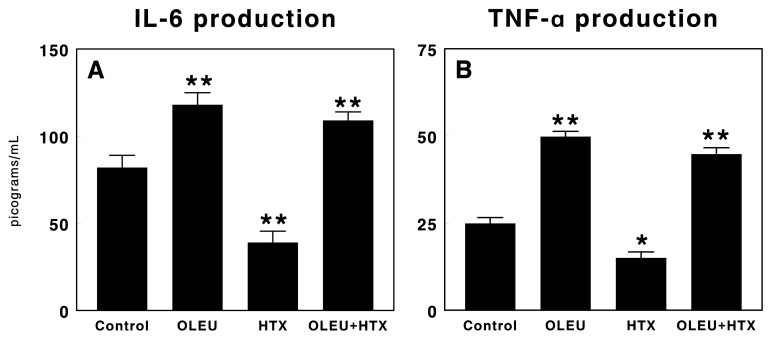
Interleukin-6 (IL-6) (**A**) and tumor necrosis factor α (TNF-α) (**B**) production in sera of animals with C6 glioma implanted into the subcutaneous region, treated with vehicle (Control), 100 µg/d oleuropein (OLEU), 100 µg/da hydroxytyrosol (HTX) and 100 µg oleuropein plus 100 µg of hydroxytyrosol/d (OLEU + HTX) for five days. Results are expressed in picograms per mL (Mean ± SEM; *n* = 8; * *p* < 0.05; ** *p* < 0.01).

Figure 3 shows the effects of the administration of oleuropein, hydroxytyrosol and a mixture of both compounds for five days on tumor growth in our experimental glioma model on the pro-inflammatory cytokines IL-6 and TNF-α production. Oleuropein administration significantly increased (*p* < 0.01) both IL-6 (Figure 3A) and TNF-α (Figure 3B), whereas hydroxytyrosol administration significantly decreased (*p* < 0.01) the production of both cytokines when compared with vehicle-treated animals or with animals treated with the mixture of oleuropein plus hydroxytyrosol. Finally, the mixture of oleuropein plus hydroxytyrosol also significantly increased both IL-6 and TNF-α production when compared with vehicle-treated animals or animals treated with hydroxytyrosol, although no differences were found when compared with animals treated with oleuropein alone (Figure 3).

## 4. Discussion

RAS activation is classically related to an increase in the inflammatory status characterized by the induction of the expression of several cytokines and chemokines, transcription factors and cell adhesion molecules [3,4]. Most of the pro-inflammatory effects are mediated by the binding of AngII to AT_1_ receptor, although AT_2_ receptor activation is also involved in some inflammatory processes. AngII can also produce large amounts of proinflammatory cytokines such as TNF-α, IL-1 and IL-6 [21]. In contrast, more recent studies point to the role of Ang1-7/Mas/ACE-2 as an important anti-inflammatory axis [22]. We have previously described that the RAS is a putative key cascade in glioma tumor development [10] through the increase in ASAP activity and the decrease in APN/APB activities. These changes suggested that the metabolism of AngII to AngIII is constant, whereas the metabolism of AngIII to AngIV is slow, resulting in the predominant actions of AngII and AngIII in the animal model of C6 glioma transplanted at the subcutaneous region. Apart from their putative role promoting tumor growth, both angiotensins could also be responsible of the proinflammatory status found here, with increased levels of IL-6 and TNF-α, acting through their angiotensin receptors. In a previous report using this experimental glioma model [20], we also described that hydroxytyrosol treatment decreased the tumor growth, whereas the treatment with oleuropein or oleuropein plus hydroxytyrosol did not have any effect on tumor growth or even increases it, as occurs with oleuropein. Here, we also found that hydroxytyrosol administration also promotes an anti-inflammatory status, decreasing the levels of IL-6 and TNF-α production under the levels of untreated control animals. Furthermore, this anti-inflammatory status is not observed when oleuropein alone o in combination with hydroxytyrosol is administrated. Therefore, the antitumor effect found for hydroxytyrosol is accompanied by an anti-inflammatory effect. The inhibitory effects of hydroxytyrosol administration on tumor growth against several tumor cell lines have been also described in a growing number of studies, likely involving inactivation of the AKT and nuclear-factor-kappa B (NFκB) [23] or other pathways [24,25]. In vivo, hydroxytyrosol seems to induce cell cycle arrest and apoptosis [26,27].

**Figure 4:**
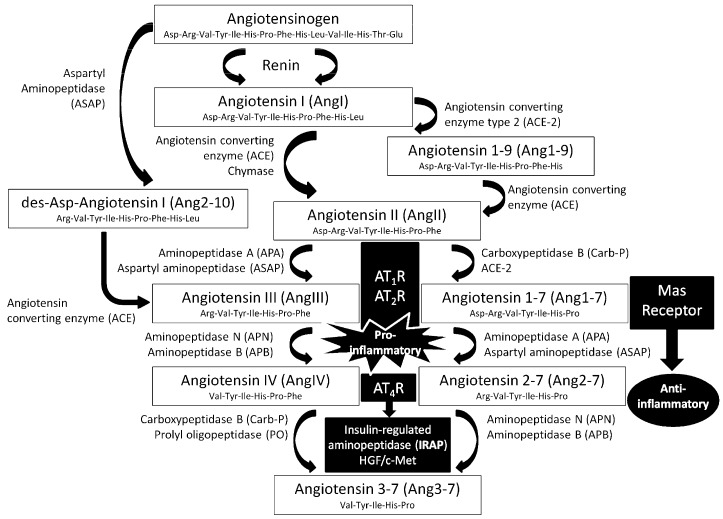
Angiotensin peptides are derived from the precursor protein angiotensinogen through several enzymatic conversion pathways. The decapeptide angiotensin I (AngI) is formed by renin acting upon the amino terminal of angiotensinogen. AngI serves as a substrate for angiotensin converting enzyme-1 (ACE-1), a zinc metalloprotease that hydrolyzes the carboxyl terminal dipeptide His-Leu to form the octapeptide angiotensin II (AngII). AngII is converted to the heptapeptide angiotensin III (AngIII) by aminopeptidase A (APA) and aspartyl aminopeptidase (ASAP), which cleaves the Asp residue at the N-terminal. Aminopeptidase N (APN) and/or aminopeptidase B (APB) cleaves Arg at the N-terminal of AngIII to form the hexapeptide angiotensin IV (AngIV). AngIV can be further converted to inactive peptide fragments and amino acid constituents. Angiotensins exert their actions through the different angiotensin receptor subtypes AT_1_, AT_2_ and AT_4_. The AT_4_ receptor has been proposed to be the insulin-regulated aminopeptidase (IRAP) or the hepatocyte growth factor (HGF)/c-Met receptor system. Other conversion steps are also shown. A separate pathway for the synthesis of AngIII, independent of AngII formation, is via the nonapeptide [des-Asp1]AngI, formed from AngI by ASAP. This nonapeptide is converted directly to AngIII via ACE-1. A third route involves the ACE-1 homolog ACE-2, which promotes AngII degradation to the heptapeptide Ang1-7. The effects of Ang1-7 are mediated through the Mas receptor. ASAP and APA inactivate Ang1-7, converting it into Ang2-7. Whereas AngII/AngIII promote a pro-inflammatory response through both AT_1_ and AT_2_ receptors, the ACE-2/Ang1-7/Mas cascade is involved in anti-inflammatory effects. The role of AngIV in inflammation remains unclear.

Regarding the RAS, here we found that oleuropein highly increased all the RAS-regulating activities measured, promoting a cascade deregulation which could be responsible of the enhanced effect of oleuropein on tumor growth, or at least, of its inefficiency to inhibit it. On the other hand, hydroxytyrosol, which clearly inhibited tumor growth in our in vivo glioma model, only modified ASAP and IRAP activities, increasing them. The changes in ASAP activity suggest an increased metabolism of AngII to AngIII, but also an increased inactivation of Ang1-7. Also, the changes in IRAP activity suggest an increased inactivation of AngIV. As a result, an increase in AngIII and a decrease of Ang1-7 could be the resulting scenario in which glioma tumor growth is inhibited by hydroxytyrosol. In fact, AngII and expression of AT1 and AT2 receptors are associated with a high grade of malignancy, increased cellular proliferation and angiogenesis [7], whereas AngIII could have the opposite effects. However, Ang1-7/Mas signaling has been shown to inhibit the growth and invasiveness of several human cancers including glioblastoma [28], which is not in agreement with our results. In the same sense, Ang1-7/Mas axis has been proposed as a counter-regulator system to de deleterious effects of AngII/AT_1_ receptor activation [22], which is also opposed to the anti-inflammatory status promoted by hydroxytyrosol in this experimental model. It could be possible that the interaction between the different RAS-regulating aminopeptidase activities promotes levels of angiotensin peptides different to those initially proposed (Figure 4). Therefore, further research is necessary to clearly evaluate the relationship between inhibitory effects of hydroxytyrosol on tumor growth and the Ang1-7/Mas receptor signaling cascade, evaluating the levels of the different angiotensins to clearly correlate them with their corresponding regulating aminopeptidase specific activities.

Interestingly, we have also described here that oleuropein alone or in combination did not modify tumor growth in this animal model and that even promotes a pro-inflammatory status increasing IL-6 and TNFα production. However, the antioxidant capacity of oleuropein has been demonstrated to be higher than of hydroxytyrosol. In fact, oleuropein has been described as a potent scavenger of oxygen free radicals [29] and nitrogen-based free radical species [30]. In addition, it plays an important role in the prevention of DNA damage, thus inhibiting mutagenesis and carcinogenesis [31]. Furthermore, it has been demonstrated that the antitumor effect of oleuropein may be exerted by the disruption of actin filaments in tumor cells [32]. In the same way, oleuropein has been described as an anti-inflammatory compound [33,34]. However, to date, the antitumor effects of oleuropein and hydroxytyrosol have mainly been described by in vitro studies. Some in vivo studies have also found antiproliferative effects for oleuropein [27]. Therefore, the effects of oleuropein remain controversial, and could be related to the different concentration/doses administrated and/or the administration routes used. In fact, previous experiments of our group using higher doses of oleuropein promoted a stimulatory effect on tumor growth in this animal model (data not shown). Therefore, the mechanisms underlying the stimulatory effects of oleuropein on tumor growth found in our previous studies remain to be resolved, but could be related to the pro-inflammatory effects found here.

Our results also show that the in vivo administration of a mixture of both compounds is inefficient to both prevent tumor development and promote an anti-inflammatory status, suggesting the opposite functions of oleuropein and hydroxytyrosol. The in vivo effects of hydroxytyrosol are highly dependent on the dose used [35], and some competition between both compounds can occur, resulting in the blocking of the effects of hydroxytyrosol. In any case, the effects of these phenolic compounds are clearly not only related to their antioxidant properties and may also modulate additional processes such as those regulated by the RAS and inflammation, among others.

## 5. Conclusions

We can conclude that a relation exists between RAS-regulating aminopeptidase activities, tumor growth and pro-inflammatory status in our glioma model in vivo, and that the inhibitory effects on tumor growth and the anti-inflammatory status promoted by hydroxytyrosol, but not by oleuropein or by the mixture of oleuropein plus hydroxytyrosol, clearly indicates that these effects are related not only with their antioxidant properties, but also with their influence on different metabolic pathways/cascades such as those mediated by the RAS probably acting through AngIII and Ang-1-7 and other related to the regulation of inflammatory processes.
